# Perioperative Strategies to Reduce Occupational HIV Exposure in Head and Neck Microsurgical Reconstruction: A Case Report

**DOI:** 10.7759/cureus.91460

**Published:** 2025-09-02

**Authors:** Tatsuya Ishii, Naoki Matsuura, Ryo Fukuda, Edward H Ntege, Yusuke Shimizu

**Affiliations:** 1 Plastic and Reconstructive Surgery, University of the Ryukyus Hospital, Ginowan, JPN

**Keywords:** free tissue flaps, head and neck neoplasms, hiv infections, infection control, microsurgery, needlestick injuries, occupational exposure, tongue neoplasms

## Abstract

Complex head and neck reconstructive surgeries in patients living with human immunodeficiency virus (HIV) present unique risks of occupational bloodborne pathogen exposure. We report the case of a 63-year-old man with well-controlled, long-standing HIV infection who underwent hemiglossectomy and microvascular free flap reconstruction for advanced tongue cancer. He was receiving antiretroviral therapy and had a CD4^+^ T-cell count of 361 cells/µL-above the threshold for acquired immunodeficiency syndrome (AIDS)-consistent with partial immune recovery. To minimize occupational exposure risk, we implemented a structured, procedure-specific exposure reduction bundle tailored to intraoral surgery. This included instrument-only suturing facilitated by an intraoral mouth opener, extended-length needle holders to increase working distance from the patient’s dentition, a predefined hands-free neutral zone with a designated needle parking area for instrument exchange, and readiness to initiate post-exposure prophylaxis. An anterolateral thigh free flap was harvested and inset without intraoperative complications. Notably, no occupational exposures occurred. The flap remained viable, and the patient was discharged on postoperative day 35. This framework may serve as a practical reference for surgical teams managing similar high-risk intraoral procedures in patients with HIV.

## Introduction

Modern antiretroviral therapy (ART) has markedly improved the prognosis of people living with human immunodeficiency virus (HIV), with contemporary cohort data from high-income settings showing near-normal life expectancy in many contexts [1]. In parallel, current perioperative guidance advises that indicated surgery should not be denied or delayed solely on the basis of HIV status; routine preoperative assessment is appropriate as for any other patient, and emergency or urgent procedures should not be postponed to obtain CD4^+^ T-cell counts or viral load results [2,3].

Head and neck procedures, particularly intraoral and oropharyngeal reconstruction, combine a confined, three-dimensional workspace, sharp dentition, limited visibility, and frequent instrument exchanges, all of which increase opportunities for bloodborne pathogen exposure. The average risk of HIV transmission after a percutaneous injury is commonly cited at approximately 0.2%-0.3% (about 2-3 per 1,000 exposures), with substantially lower risk from mucocutaneous splashes; timely, guideline-concordant post-exposure prophylaxis (PEP), initiated as soon as possible and no later than 72 hours, further mitigates this risk [4-10]. Operational factors, such as rapid access to PEP, adherence, and completion of the full course, also influence uptake and staff confidence [6,7]. In this report, instrument-only suturing refers to handling suture needles exclusively with instruments (no finger-near-needle manipulation), and a hands-free neutral zone denotes a clearly visible surface designated for placing and retrieving sharps between team members [8]. At a population level, needlestick injuries remain a persistent hazard for healthcare workers worldwide, underscoring the need for context-specific safeguards in the operating room [11].

The anterolateral thigh (ALT) free flap remains a workhorse for head and neck reconstruction owing to its reliable anatomy, tissue versatility, and acceptable donor site profile. Recent syntheses comparing ALT with radial forearm free flaps in oral cavity reconstruction report equivalent flap survival and oral function with generally lower donor site morbidity for ALT, and head-and-neck-focused reviews continue to support favorable donor site and aesthetic outcomes [12-14]. Despite these advantages, intraoral inset typically demands meticulous suturing and prolonged operative time-conditions that can amplify exposure opportunities, especially when viremia is not suppressed. Concurrently, perioperative safety literature supports measures such as double-gloving, use of blunt-tip suture needles where appropriate, and hands-free neutral-zone passing to reduce glove perforation and sharps-injury risk [11,15,16].

Here, we describe the perioperative management of a patient with HIV undergoing hemiglossectomy with microvascular free flap reconstruction, emphasizing a structured, procedure-specific exposure reduction bundle spanning the intraoperative and postoperative phases. Given the extended duration of intraoral microsurgery and the frequency of needle and instrument exchanges, safeguards beyond standard precautions are warranted. By detailing practical, reproducible measures associated with a zero-exposure outcome during prolonged oral-cavity reconstruction, we address a key implementation gap for plastic and reconstructive surgery teams managing similar high-risk cases.

## Case presentation

A 63-year-old Japanese man presented to our Plastic and Reconstructive Surgery department with a painful mass on the left lateral tongue. Nine years earlier, he was diagnosed with HIV infection and progressed to acquired immunodeficiency syndrome (AIDS) after *Pneumocystis jirovecii* pneumonia. He immediately began combination ART with dolutegravir, tenofovir disoproxil fumarate, and emtricitabine. Later that same year, he developed diffuse large B-cell lymphoma treated with five cycles of cyclophosphamide, doxorubicin, vincristine, and prednisone (CHOP). He also received treatment for syphilis with intravenous (IV) aqueous penicillin G, 18 million units per day for 14 days, and for cytomegalovirus-associated adrenal insufficiency with corticosteroids (hydrocortisone 300 mg/day IV, tapered to 20 mg/day over seven days and then maintained). At the time of his AIDS diagnosis, CD4^+^ and CD8^+^ T-cell counts were 47 and 253 cells/µL, respectively, and plasma HIV ribonucleic acid (RNA) was 138,000 copies/mL. Six months after initiating ART, HIV RNA became undetectable and has remained suppressed.

**Figure 1:**
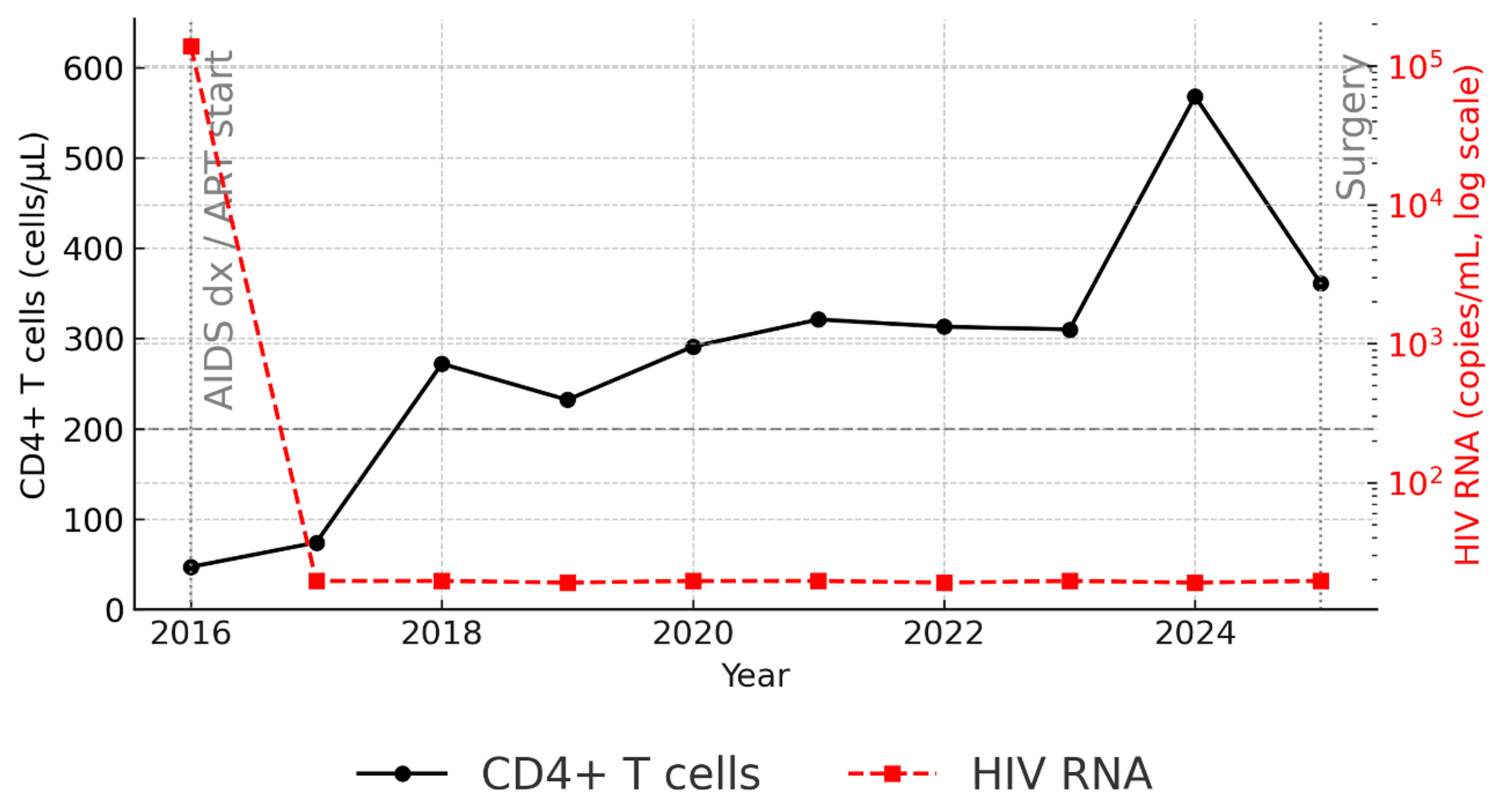
Longitudinal immune and virologic trends Black solid line (left y-axis) shows CD4^+^ T-cell counts; red dashed line with square markers (right y-axis, base-10 logarithmic scale) shows plasma HIV RNA levels. ART initiation in 2016 was followed by durable viral suppression (“undetectable,” i.e., below the assay’s lower limit of quantification, <20 copies/mL; log value ≲ 1.3) and gradual CD4^+^ recovery above 300 cells/µL prior to surgery in 2025. Vertical dotted lines mark key clinical events (AIDS diagnosis/ART start; surgery). A horizontal dashed line at 200 cells/µL denotes the AIDS threshold. This figure demonstrates sustained virologic control with adequate immune reconstitution before major surgery. ART: antiretroviral therapy; AIDS: acquired immunodeficiency syndrome; HIV: human immunodeficiency virus; RNA: ribonucleic acid

From 2016 through 2025, he underwent systematic annual evaluations at our infectious disease clinic in accordance with national guidelines [17]. Testing included immunologic (CD4^+^/CD8^+^ T-cell counts), virologic (HIV RNA), and serologic screening for hepatitis viruses, syphilis, and other infections, performed at a single certified laboratory with consistent reference ranges. As part of the 2025 preoperative assessment, updated hepatitis panels and syphilis serologies (rapid plasma reagin (RPR) and *Treponema pallidum* latex agglutination (TPLA)) were obtained for surgical risk evaluation. Consolidated longitudinal results are summarized in Table 1, and Figure 1 depicts immune recovery with sustained virologic suppression supporting surgical candidacy.

**Table 1:** Longitudinal HIV-related laboratory parameters (2016–2025) CD4^+^ increased from 47 (2016) to a peak of 568 (2024) with a preoperative value of 361 (2025), while HIV RNA was suppressed at <20 copies/mL from 2017 onward, supporting immunologic stability prior to surgery. ^†^Undetectable indicates below the assay lower limit of quantification (LLOQ), <20 copies/mL. ART: antiretroviral therapy; PCP: *Pneumocystis jirovecii* pneumonia; CMV: cytomegalovirus; HBsAg: hepatitis B surface antigen; HBsAb: hepatitis B surface antibody; HBcAb: hepatitis B core antibody; HCV Ab: hepatitis C virus antibody; HTLV-1 Ab: human T-cell leukemia virus type 1 antibody; RPR: rapid plasma reagin; TPLA: *Treponema pallidum* latex agglutination; ND: not determined; AIDS: acquired immunodeficiency syndrome; HIV: human immunodeficiency virus; RNA: ribonucleic acid

Year	Age (year)	Clinical event/note	CD4^+^ (cells/µL)	CD8^+^ (cells/µL)	HIV RNA (copies/mL)	HBsAg (IU/mL)	HBsAb (mIU/mL)	HBcAb (S/CO)	HCV Ab (S/CO)	HTLV‑1 Ab (C.O.I)	RPR (RU)	TPLA (TU)
2016	54	AIDS diagnosis; start ART; PCP; CMV adrenal insufficiency; syphilis	47	253	138,000	Negative	41.5	6.5	0.1	Positive	162.0	4,270
2017	55	Pre-op year −8	74	246	<20	ND	ND	ND	ND	ND	45.7	1,030
2018	56	Pre-op year −7	272	932	<20	ND	ND	ND	ND	ND	27.3	619
2019	57	Pre-op year −6	232	622	Undetectable^†^	ND	ND	ND	ND	ND	23.5	475
2020	58	Pre-op year −5	291	642	<20	ND	ND	ND	ND	ND	16.1	396
2021	59	Pre-op year −4	321	627	<20	ND	ND	ND	ND	ND	11.2	342
2022	60	Pre-op year −3	313	599	Undetectable^†^	ND	ND	ND	ND	ND	9.8	316
2023	61	Pre-op year −2	310	245	<20	ND	ND	ND	ND	ND	8.7	254
2024	62	Pre-op year −1 (routine follow-up)	568	424	Undetectable^†^	ND	ND	ND	ND	ND	9.0	ND
2025	63	Preoperative assessment for tongue cancer surgery	361	285	<20	Negative	24.8	5.2	0.1	Positive	ND	208
Reference ranges	-	-	500–1,200	300–1,000	<20	<0.05	<10.0	<1.0	<1.0	<1.0	<10	<10

Presenting symptoms included oral bleeding and weight change; exposure history (tobacco, alcohol) was recorded. Intraoral examination demonstrated an ulcerated lesion on the left lateral tongue with focal erythema/leukoplakia, a firm, indurated base with surface exudate, and preserved tongue mobility; no trismus was observed. Cranial nerve (CN) examination revealed no focal deficits of CN V, VII, IX, X, or XII. Neck examination identified a solitary level IIA lymph node, approximately 20 mm in greatest dimension. Airway assessment showed adequate mouth opening and no signs of impending compromise.

**Figure 2:**
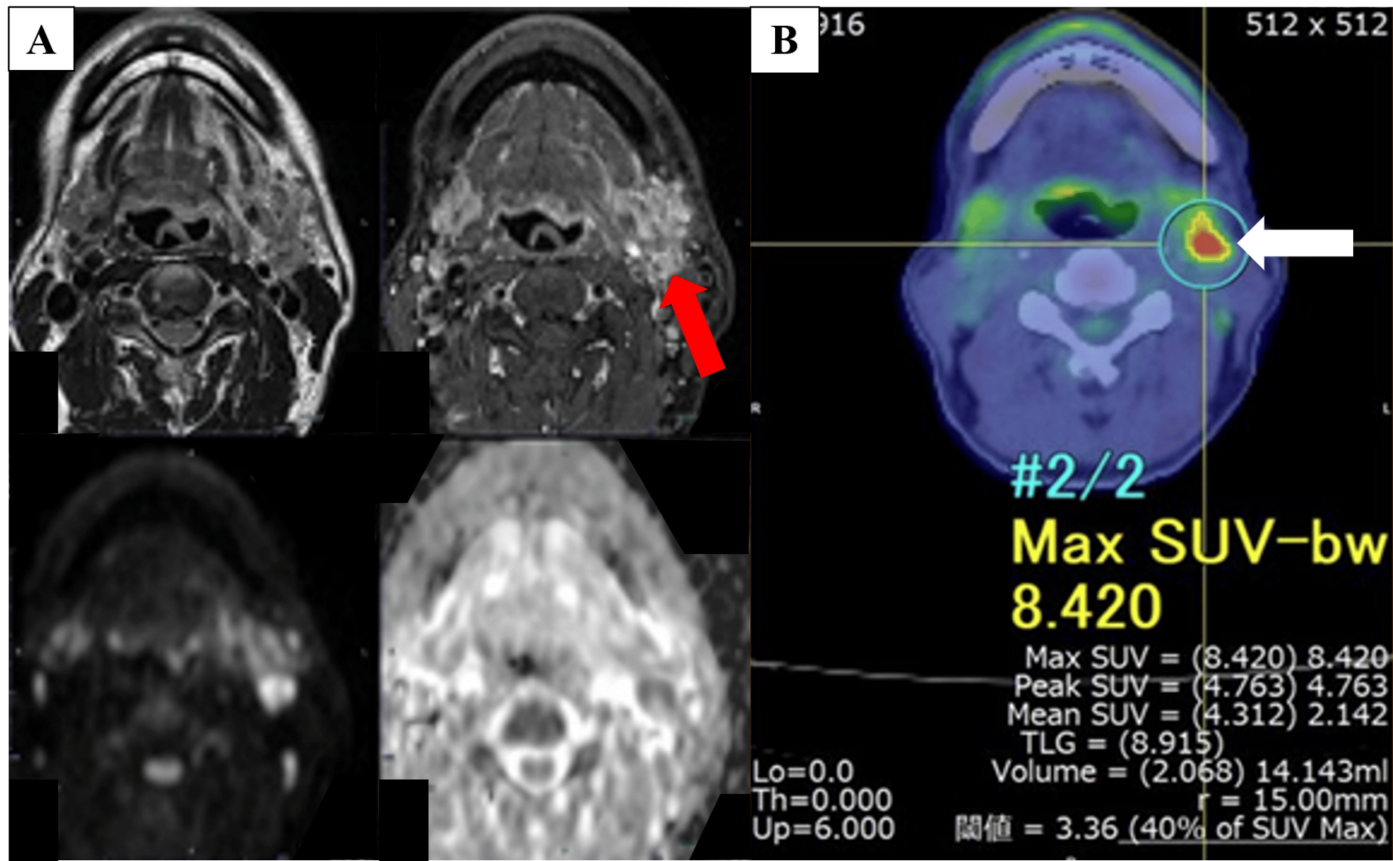
Radiologic evaluation of left tongue cancer (A) T2-weighted MRI demonstrating a hyperintense lesion (bright on T2; red arrow) at the left tongue margin with contrast enhancement and restricted diffusion, extending toward the lingual septum. (B) PET-CT revealing FDG uptake (increased glucose-tracer uptake indicating high metabolic activity) at the primary tumor and the ipsilateral cervical lymph node (white arrow). R/L orientation and scale bars are included where applicable. The imaging supports a metabolically active primary tongue malignancy with nodal involvement. MRI: magnetic resonance imaging; PET-CT: positron emission tomography-computed tomography; FDG: fluorodeoxyglucose

Magnetic resonance imaging (MRI) was obtained to delineate lingual-septum invasion and perilesional soft-tissue planes; positron emission tomography-computed tomography (PET-CT) was used for metabolic staging and nodal assessment (Figure 2). MRI showed a 17 × 22 × 41 mm T2-hyperintense lesion extending toward the lingual septum with diffusion restriction and contrast enhancement. PET-CT demonstrated fluorodeoxyglucose (FDG) uptake at the primary site and in the ipsilateral level IIA lymph node.

**Figure 3:**
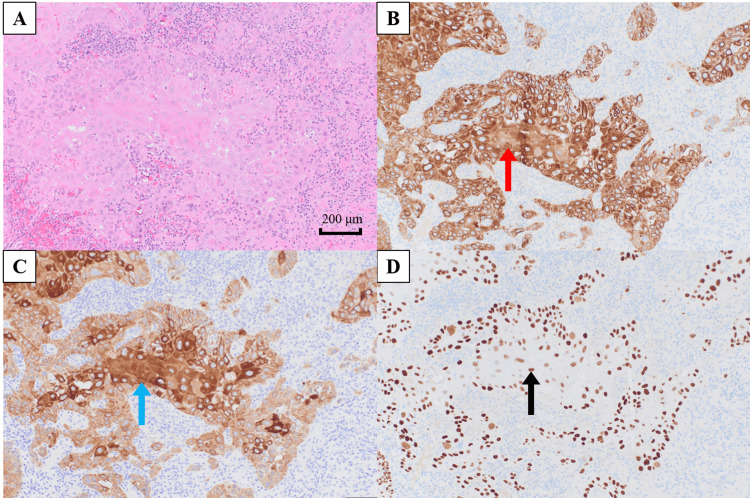
Histopathology and immunohistochemistry of the tongue lesion (A) Hematoxylin and eosin staining showing infiltrative, keratinizing squamous cell carcinoma (×100). (B) Strong, diffuse cytoplasmic positivity for cytokeratin (CK) AE1/AE3 (red arrow; ×100). (C) Cytoplasmic and membranous positivity for CK5/6 (blue arrow; ×100). (D) Strong nuclear staining for p40 confirming squamous lineage (black arrow; ×100). Scale bar = 200 µm. At ×100, cellular architecture and squamous differentiation features are appreciable; the combined morphology and immunophenotype support SCC. SCC: squamous cell carcinoma

Incisional biopsy confirmed infiltrative, keratinizing squamous cell carcinoma (SCC) (Figure 3A). Immunohistochemistry showed strong, diffuse cytoplasmic AE1/AE3 positivity, cytoplasmic and membranous cytokeratin (CK) 5/6 positivity, and strong nuclear p40 staining, consistent with squamous differentiation (Figures 3B-3D). Clinical staging was cT4aN1M0 (stage IVA) per the American Joint Committee on Cancer (AJCC) eighth edition [10]. The differential diagnosis for a painful lateral-tongue mass in a person with well-controlled HIV included neoplastic lesions (oral-cavity SCC, lymphoma, and Kaposi sarcoma), infectious/inflammatory etiologies (traumatic ulcer, candidiasis, aphthous ulcer, and syphilitic ulcer), and other mucosal lesions; serology and examination did not suggest active syphilis.

**Figure 4:**
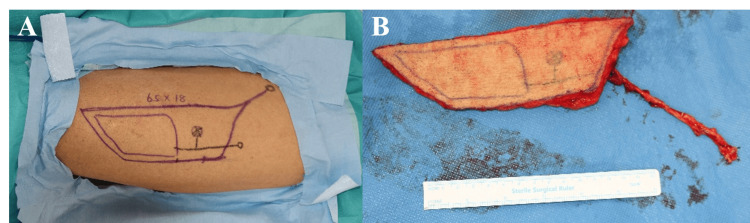
ALT (anterolateral thigh) flap design and harvest (A) Preoperative skin markings for a 6.5 × 18 cm ALT flap on the left thigh. (B) Harvested flap with a musculocutaneous perforator identified preoperatively by duplex ultrasonography, arising from the descending branch of the lateral circumflex femoral artery. The ALT is a commonly used soft-tissue flap for tongue reconstruction due to its reliable anatomy and adequate bulk.

Under general anesthesia, the patient underwent hemiglossectomy, ipsilateral neck dissection (levels I-IV), and tracheostomy. Reconstruction was performed using a 6.5 × 18 cm left ALT flap designed with preoperative duplex ultrasonography, which identified a musculocutaneous perforator from the descending branch of the lateral circumflex femoral artery (Figures 4A, 4B). Microvascular anastomoses to cervical recipient vessels were performed under the operating microscope.

Before incision, the team conducted a focused hazard review (“time-out plus”) to identify intraoral exposure risks-proximity to dentition, a confined three-dimensional workspace, and frequent instrument exchange. A standardized set of distance-increasing and handling measures was then applied: (i) a neck-field suturing route whenever feasible, reserving intraoral access only when required (tongue and ALT inset), (ii) mouth retractors and extended-length needle holders to enable instrument-only (“mechanical”) suturing at an increased operator-dentition distance (no finger-near-needle manipulation), and (iii) a predefined hands-free neutral zone on the Mayo stand for all needle-bearing instrument exchanges, with systematic needle parking between throws. Intraoperative debriefing indicated strong user acceptance: nurses described the workflow as “clear and safe due to the neutral zone for instrument exchange,” and surgeons noted that the long-handled needle holder reduced contact with the teeth. These measures were selected and rehearsed during the preoperative briefing and referenced during inset to maintain adherence. Donor site closure was performed with continuous nylon sutures rather than staples.

**Figure 5:**
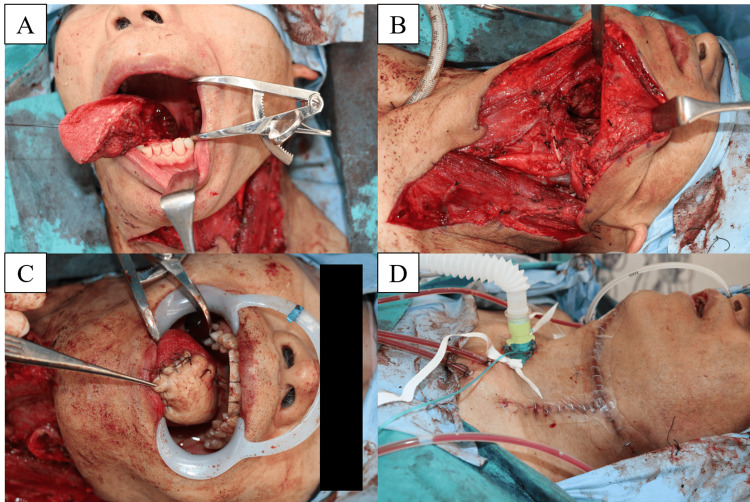
Intraoral reconstruction and occupational HIV exposure reduction measures (A) Post-hemiglossectomy intraoral defect prior to inset. (B) Distance-increasing setup: mouth retractors and long needle holders maintain hand distance from dentition. (C) Flap inset using a hands-free neutral-zone instrument-exchange protocol (neutral-zone area indicated) with instrument-only (“mechanical”) suturing and needle parking between throws. (D) Final intraoral view after layered suturing and tracheostomy completion. The neutral zone is a pre-agreed, clearly visible surface for hands-free placement/pick-up of sharps; instrument-only suturing denotes needle driving exclusively with instruments (no finger-near-needle manipulation). The sequence illustrates a reproducible, procedure-specific bundle that increases operator-dentition distance and standardizes sharps handling. HIV: human immunodeficiency virus

See Table 2 for the full perioperative bundle and Figures 5A-5D for the intraoral workflow. These measures were selected to (1) increase the working distance from dentition, (2) minimize sharps handling and unplanned handoffs, and (3) standardize instrument exchange within a hands-free neutral zone.

**Table 2:** Perioperative HIV exposure reduction bundle PEP: post-exposure prophylaxis; OR: operating room; ICU: intensive care unit; neutral zone: a pre-agreed, clearly visible surface for hands-free placement/pick-up of sharps; needle parking: placing the needle in the neutral zone between throws; instrument-only (mechanical) suturing: needle driving exclusively with instruments (no finger-near-needle manipulation). Measures were selected during a pre-incision hazard review and rehearsed during team briefing; adherence was reinforced intraoperatively. Nursing staff described the neutral-zone workflow as “clear and safe,” and surgeons reported reduced tooth contact with a long-handled needle holder, supporting feasibility and acceptance. Cost/feasibility: in our setting, additional resources (extended-length needle holders, neutral-zone tray or magnetic mat, and a stocked PEP starter kit) were obtained for under approximately US $100 per case.

Phase	Objective	Specific measure	Rationale	Personnel
Pre-op	Rapid PEP access	OR starter kit-bictegravir/emtricitabine/tenofovir alafenamide-and an action manual; location confirmed during team briefing.	Enable rapid initiation of PEP as soon as possible and within 72 h if exposure occurs; reduce delays.	Surgeon, scrub nurse, infectious diseases (ID) team
Intra-op (oral cavity)	Increase distance from dentition	Mouth opener, oral retractors, extended-length needle holders; instrument-only (“mechanical”) suturing.	Reduce hand-tooth proximity; reduce sharps risk.	Primary surgeon, assistant
Intra-op (instrument handling)	Eliminate hand-to-hand sharps transfer	Hands-free neutral-zone tray/instrument pass; needle parking between throws.	Decrease needlestick injuries; avoid unplanned handoffs.	Surgeon, scrub nurse, circulating (scout) nurse
Intra-op (donor site)	Limit sharps at closure/removal	Continuous nylon sutures (vs. staples); consider tissue adhesive where tension is low.	Fewer sharp removals; avoid minor wound-complication signal reported with staples.	Donor site surgeon
Post-op monitoring	Maintain distance during flap checks	22-gauge needle mounted on a 20 mL syringe for pin-prick.	Increase operator-mouth distance.	ICU/ward nurses, surgeons
Post-op suture removal	Prevent splatter/cuts	Stand contralateral; blunt scissors under gauze shielding.	Minimize splash and sharps injury.	Ward staff, residents

The total operative time was 9 hours 11 minutes with an estimated blood loss of 274 mL. The patient was monitored in the intensive care unit for 48 hours. The flap remained viable without vascular compromise or infection. No intraoperative or postoperative occupational blood-exposure events were reported. He resumed oral intake as planned and was discharged home on postoperative day 35; longer-term validated functional assessments were not yet available given the short interval since discharge.

## Discussion

This case demonstrates that complex head and neck free flap reconstruction can be performed safely in patients with HIV when a structured, procedure-specific safety bundle is applied. The patient underwent hemiglossectomy, cervical lymphadenectomy, and ALT flap reconstruction with no intraoperative complications, no postoperative infection, and no occupational exposure despite a prior AIDS diagnosis. These findings are consistent with contemporary evidence that durable virologic suppression and adequate CD4⁺ reconstitution mitigate perioperative risk and should not preclude major oncologic surgery, in line with modern survival data and perioperative guidelines [1-3].

Oral cavity reconstruction carries a disproportionate exposure risk due to the confined three-dimensional field, sharp dentition, and prolonged instrument manipulation. Although recent public health guidance indicates that HIV transmission via saliva is extraordinarily rare [18], blood contact from sharps or abrupt patient movement remains a realistic hazard. Accordingly, we increased the distance between the operator and the patient’s dentition using a mouth opener and oral retractors and performed flap inset with extended-length needle holders to enable instrument-only (“mechanical”) suturing at a safer working distance. Recent summaries place the average per-exposure HIV transmission risk after a percutaneous injury at approximately 0.2%-0.3%, with substantially lower risk for mucocutaneous splash exposures; prompt, guideline-concordant PEP further reduces this risk [4,6,10]. To further suppress sharps risk, we implemented a hands-free neutral-zone passing technique, eliminating hand-to-hand instrument transfer. This approach is endorsed by current perioperative sharps-safety guidance and is associated with fewer needlestick injuries in the operating room [8]. In our nine-hour operation, the hands-free technique was practical and contributed to a zero-exposure outcome. We also used double-gloving and full face/eye protection, consistent with contemporary recommendations for high-risk procedures [8].

Donor site closure was performed with continuous nylon sutures rather than skin staples to decrease sharps handling during removal. While a recent systematic review found no clear advantage of staples over sutures in preventing infection, it noted that staples may increase the risk of minor wound complications; in our setting, using sutures also minimized additional sharps handling during removal [19]. Tissue adhesive may be considered where wound tension is low. Our zero-exposure outcome aligns with programmatic reports of no seroconversions when occupational PEP is initiated promptly after percutaneous exposure and contrasts with the substantial global needlestick burden among healthcare workers, underscoring the value of practical, procedure-specific operating room safeguards [4,8,10].

Institutional preparedness was integral to our approach. A PEP “starter kit” (bictegravir/emtricitabine/tenofovir alafenamide) and an action manual were pre-positioned in the operating room to enable rapid initiation of therapy-as soon as possible and within 72 hours of exposure-consistent with peer-reviewed national guidance and recent international recommendations to expand PEP accessibility [6,9]. While some centers maintain similar readiness, detailed procedure-level descriptions of such protocols remain uncommon in the surgical literature for patients with HIV. Postoperative protocols were likewise adapted to maintain distance and reduce sharps handling. Flap monitoring was performed using a 22-gauge needle mounted on a 20 mL syringe rather than a handheld needle, and suture removal was done from the contralateral side with blunt scissors under gauze shielding to minimize splatter and injury risk-a prudent approach given the known occupational risk profile and the importance of timely PEP initiation after any suspected exposure [6,10].

The patient’s preoperative CD4⁺ count of 361 cells/µL with consistently undetectable HIV RNA exceeded thresholds commonly used to support elective surgery in patients with HIV (e.g., CD4 > 200 cells/µL with viral suppression), reinforcing the safety and appropriateness of proceeding with microvascular reconstruction in this context [2,3]. Indeed, given the patient’s sustained immune recovery and virologic control, the benefits of curative surgical extirpation outweighed the risks, especially with our enhanced precautions in place [1-3].

Existing reports in HIV-positive head and neck cancer populations tend to emphasize oncologic or general perioperative outcomes but rarely delineate a reproducible, procedure-specific intraoperative exposure reduction bundle. Our protocol (Table 2) integrates mechanical (distance-increasing instrumentation, instrument-only suturing, and neutral-zone handoffs), behavioral (team briefings, staff positioning), and pharmacologic (PEP readiness) safeguards across the perioperative continuum, aligning with current sharps-safety guidance and implementation literature [8].

Because the bundle is anchored to site-agnostic principles-maintain distance, standardize hands-free sharps exchange via a neutral zone, and ensure rapid PEP access-it can be adapted by substituting specialty-specific instruments and positioning the neutral zone within the sterile field. For example, wound protectors or ring retractors (general/colorectal), long laparoscopic needle drivers (minimally invasive surgery), wire twisters and K-wire caps (orthopedics), weighted specula and long needle holders (vaginal surgery), long bayonet instruments (neurosurgery), and wire-management strategies (cardiothoracic) serve the same function as our oral retractors and extended-length needle holders-they increase working distance and reduce unplanned handoffs. Current perioperative guidance endorses hands-free neutral-zone passing to minimize sharps injuries, and checklist-based implementation resources support cross-specialty standardization; choosing a clearly visible, stable surface (e.g., the corner of a Mayo stand, a magnetic mat, or a tray) and using “needle parking” between suture throws help maintain reproducibility across services [11,20]. We provide practical examples by specialty in the Appendix to facilitate local customization and training.

In addition to reducing exposure risk, visible preparedness (a stocked PEP kit and action card in the operating room) and a clearly defined neutral-zone workflow may lessen staff anxiety by making the response pathway immediate and transparent. In our case debrief, frontline feedback indicated strong user acceptance-nurses described the neutral-zone workflow as “clear and safe,” and surgeons noted that long-handled needle holders reduced the chance of tooth contact. To support sustained adherence, we propose brief educational measures (a pre-incision briefing to establish the neutral zone, short micro-drills for instrument-only suturing and needle parking, and scenario-based PEP activation simulations) paired with light-touch process metrics (near-miss and adherence logs, plus brief staff confidence checks), and we will track simple process measures-for example, the proportion of needle passes via the neutral zone, documented needle parking, and use of distance-increasing instruments-during subsequent cases and iteratively refine the bundle through plan-do-study-act cycles [11,20]. Embedding these steps into routine practice could strengthen safety culture and provider well-being alongside clinical outcomes.

Required additional resources, including extended-length needle holders, a neutral-zone setup, and a stocked PEP kit, were inexpensive in our setting and required minimal training, making this bundle readily adaptable in both resource-rich and resource-limited environments. Given the ongoing global burden of needlestick injuries among healthcare workers, implementing such standardized measures may provide system-level benefits [11].

This single-institution experience cannot establish causality or broad generalizability. Multicenter, prospective evaluations with pre-post implementation comparisons are warranted and should specify a priori outcomes such as exposure events per 1,000 operative hours, time-to-PEP initiation, and audited adherence. Future work should also evaluate simulation-based training, enhanced barrier devices, and digital auditing of sharps handling to further reduce exposure risk and adapt protocols for patients with high viral loads or incomplete immune reconstitution. The current report reflects a short postoperative interval; longer-term outcomes (e.g., oncologic control, late complications, and validated functional recovery at 3-12 months) were not available at the time of writing. We lacked a pre-implementation comparator, and adherence to the bundle was assessed qualitatively, introducing potential Hawthorne and self-reporting effects. Generalizability may also be constrained by local workflow and case mix.

## Conclusions

This case demonstrates that complex head and neck free flap reconstruction can be performed safely in a patient with well-controlled HIV when a structured exposure reduction protocol is used. The patient’s hemiglossectomy and ALT flap reconstruction were completed without intraoperative complications or occupational blood exposures. Key elements included distance-increasing instrumentation (e.g., extended-length tools and instrument-only suturing), hands-free neutral-zone exchange to eliminate hand-to-hand sharps transfers, and immediate availability of PEP. These precautions align with contemporary sharps-safety practices and perioperative HIV care guidance.

The protocol was low-cost, required minimal additional training, and integrated smoothly into the surgical workflow, suggesting potential applicability across teams and settings. Because this is a single case with limited follow-up, the findings are preliminary. Multicenter, pre-/post-implementation evaluations with 3-12-month clinical, functional, and staff-safety outcomes are needed to establish efficacy and refine the approach. If validated, wider adoption of procedure-specific safeguards may improve occupational safety while maintaining high-quality oncologic reconstruction for patients living with HIV.

## Appendices

**Table 3:** Adaptation of the exposure reduction bundle by surgical specialty Each row lists distance-increasing instrument swaps, hands-free neutral-zone placement strategy (including needle parking), and the specific sources supporting those recommendations. “Instrument-only suturing”: needle driving exclusively with instruments (no fingers near the needle); “neutral zone”: pre-agreed, clearly visible surface used for hands-free placement/pick-up of sharps; “needle parking”: placing needle-bearing tools in the neutral zone between throws to prevent unplanned handoffs

Specialty/setting	Distance-increasing instrument swaps (examples)	Neutral-zone placement & hands-free passing (examples)	References
General/colorectal (open)	Wound protector or ring retractor; long needle drivers; long Metzenbaum scissors; instrument-only suturing for deep fascia when feasible; blunt-tip suture needles for fascia.	Neutral zone on Mayo‑stand corner or magnetic mat; verbal call-outs (e.g., “needle to zone”); needle parking between throws.	[9,20,21]
Minimally invasive (laparoscopy/robotics)	Long laparoscopic needle drivers; laparoscopic grasper for extracorporeal knot manipulation; trocar safety devices.	Neutral zone on the back table/Mayo (outside the camera field); pass trocar-related sharps to the zone (not hand-to-hand); park suture needles on tray prior to retrieval.	[9,22,23]
Orthopedics	Wire twister; long needle holders; K-wire caps; double-gloving (indicator under-glove optional).	Neutral zone on Mayo or magnetic pad; park K-wires/needles before exchange; avoid hand-backs of wire tips.	[9,20,23]
OB/GYN (open/vaginal)	Weighted speculum; ring retractors; long needle holders for deep pelvic work; blunt-tip suture needles for uterine/fascial closure.	Back-table neutral zone; announced placement/pick-up; park needle drivers between throws.	[9,21]
Cardiothoracic	Long needle drivers; dedicated wire twisters; wire capping/bending aids; double-gloving for prolonged wire handling.	Neutral zone on Mayo; wire ends secured/parked before exchange; verbal cue prior to pick-up.	[9,24]
Neurosurgery	Micro-long needle holders; tubular retractors; microscope/endoscope ports used as distance aids.	Small-tray neutral zone near the scrub with clear line of sight; one-at-a-time passing; needle counters with parking.	[9,22,25]
Endoscopy/interventional radiology	Bite block (upper GI); long injection/hemostasis tools; sheath- or introducer-based delivery to reduce hand proximity.	Procedure-cart neutral zone; place sharps on tray (not hand-to-hand); immediate sharps container use.	[22,23,26]
Head & neck (beyond oral cavity)	Mouth props/cheek retractors; splash shields; long needle holders; instrument-only suturing where feasible.	Mayo-stand neutral zone with clear line of sight; park needle drivers between throws; verbal call-outs for placement/pick-up.	[9,22]

## References

[1] Trickey A, Sabin CA, Burkholder G (2023). Life expectancy after 2015 of adults with HIV on long-term antiretroviral therapy in Europe and North America: a collaborative analysis of cohort studies. Lancet HIV.

[2] (2025). New York State Department of Health AIDS Institute. Perioperative care in adults with HIV. https://www.hivguidelines.org/guideline/hiv-perioperative/.

[3] Shin SJ, Vail RM, Shah SS (2024). Perioperative Care in Adults With HIV. https://www.ncbi.nlm.nih.gov/books/NBK576018/.

[4] Abadie RB, Brown EM, Campbell JR (2024). Incidence and risks of HIV infection, medication options, and adverse effects in accidental needle stick injuries: a narrative review. Cureus.

[5] Pierce A (2024). Management of occupational exposure to blood and body fluids in primary care. Aust Prescr.

[6] WHO WHO (2025). World Health Organization. Guidelines for HIV Post-Exposure Prophylaxis. Published July 22. Guidelines for HIV Post-Exposure Prophylaxis.

[7] Tanner MR, O'Shea JG, Byrd KM (2025). Antiretroviral postexposure prophylaxis after sexual, injection drug use, or other nonoccupational exposure to HIV-CDC recommendations, United States, 2025. MMWR Recomm Rep.

[8] Cresswell F, Asanati K, Bhagani S (2022). UK guideline for the use of HIV post-exposure prophylaxis 2021. HIV Med.

[9] Kyle E (2024). Sharps safety. AORN J.

[10] PDQ® Adult Treatment Editorial Board (2025). Lip and Oral Cavity Cancer Treatment (PDQ®)-Health Professional Version. PDQ Lip and Oral Cavity Cancer Treatment.

[11] Hosseinipalangi Z, Golmohammadi Z, Ghashghaee A (2022). Global, regional and national incidence and causes of needlestick injuries: a systematic review and meta-analysis. East Mediterr Health J.

[12] Pu JJ, Atia A, Yu P, Su YX (2024). The anterolateral thigh flap in head and neck reconstruction. Oral Maxillofac Surg Clin North Am.

[13] Ranganath K, Jalisi SM, Naples JG, Gomez ED (2022). Comparing outcomes of radial forearm free flaps and anterolateral thigh free flaps in oral cavity reconstruction: a systematic review and meta-analysis. Oral Oncol.

[14] Wang Y, Liang C, Lin Q (2025). Donor-site morbidity and aesthetic outcomes in patients undergoing head and neck reconstruction with anterolateral thigh or latissimus dorsi flaps: a systematic review and meta-analysis. J Craniomaxillofac Surg.

[15] Rippon MG, Rogers AA, Ousey KJ (2025). Glove breach occurrence during surgical procedures: the benefits of double/indicator system gloves. J Hosp Infect.

[16] Singh KV, Walia K, Farooque K, Mathur P (2025). Double gloving for self-protection in high-risk surgeries: a systematic review and meta-analysis. Syst Rev.

[17] Study Group on HIV Infection and Hemophilia for the Development of Team-Based Care and Improvement of Healthcare Standards, AIDS Policy Research Project, Commissioned by the Ministry of Health, Labour and Welfare (2025). Guidelines for Antiretroviral Therapy-March 2025 Version [in Japanese]. March.

[18] (2025). Centers for Disease Control and Prevention. HIV occupational transmission. https://www.cdc.gov/hiv/causes/occupational-transmission.html.

[19] Cochetti G, Abraha I, Randolph J (2020). Surgical wound closure by staples or sutures?: systematic review. Medicine (Baltimore).

[20] (2025). Association of periOperative Registered Nurses (AORN). Guideline for sharps safety-evidence table. Denver, CO: AORN, Inc.

[21] O'Donnell O, Gallagher C, Chaudhary AM, Iqbal A (2024). Time to consider blunt needles for implant surgery? A systematic review and meta-analysis shows that blunt suture needles reduce glove perforation. Surgeon.

[22] AORN AORN (2025). AORN. Neutral zones: the bordered region. https://www.aorn.org/article/neutral-zones.

[23] Gomaa A, Hughes S, Afanuh S (2025). NIOSH Science Blog. Bloodborne pathogen exposures continue in operating room settings. Bloodborne Pathogen Exposures Continue in Operating Room Settings. NIOSH Science Blog. November.

[24] Wyss M, Kolbe M, Grande B (2023). Make a difference: implementation, quality and effectiveness of the WHO Surgical Safety Checklist-a narrative review. J Thorac Dis.

[25] (2025). World Health Organization. WHO Surgical Safety Checklist Implementation Manual. https://iris.who.int/bitstream/handle/10665/44186/9789241598590_eng_Checklist.pdf?sequence=2&isAllowed=y.

[26] Morgan R, Haslam P, McCafferty I (2024). Provision of interventional radiology services 2023. Cardiovasc Intervent Radiol.

